# Critical Reflexivity and Positionality on the Scholar–Practitioner Continuum: Researching Women’s Embodied Subjectivities in Sport

**DOI:** 10.3390/sports11100206

**Published:** 2023-10-19

**Authors:** Fabiana Cristina Turelli, Alexandre Fernandez Vaz, David Kirk

**Affiliations:** 1 Faculty of Kinesiology and Recreation Management, University of Manitoba, Winnipeg, MB R3T 2N2, Canada; 2 Ciencias de la Actividad Física y del Deporte, Universidad Autónoma de Madrid, 28049 Madrid, Spain; 3 Departamento de Estudos Especializados em Educação, Universidade Federal de Santa Catarina, Florianópolis 88040-900, Brazil; alexfvaz@uol.com.br; 4 School of Education, University of Strathclyde, Glasgow G1 1XQ, UK; david.kirk@strath.ac.uk; 5 School of Human Movement and Nutritional Sciences, University of Queensland, St. Lucia, Brisbane, QLD 4072, Australia

**Keywords:** self-learning, autoethnography, comfort and discomfort, practitioner–researcher role, strange the familiar

## Abstract

The sports world has many prejudices that have been converted into common sense. Some relate to the idea of athletes being strong or pretty but endowed with little intelligence. There is another view, perhaps a little more accurate, around the reification of consciousness in the name of the automation and maximum outcome of the body. Both views are informed by Cartesian thinking, perpetuating the mind–body dichotomy. Such a dichotomy is spread in several other areas in our society, expressed as binaries. We meet a binary when conducting research as well, disembodying the researcher as someone who is neutral, objective, and highly rational, and someone who, in synthesis, performs good mental work, but who must not let feelings intrude. On the contrary, we argue that we are embodied beings who are often not able to (and maybe should not) become detached from previous experiences and knowledge when conducting research. Even though this can present itself as a challenge, we consider that a fluid non-binary positioning encompasses actions holistically and leads to tasks being performed on a continuum. The purpose of this paper is to explore the reflexive process embedded in carrying out a PhD project committed to studying the production of the embodied subjectivities of a group of women high-level athletes in karate. The researcher inserted in the researched environment was not a high-level athlete; however, she had several experiences competing at the amateur level in different countries and faced experiences that were, to some extent, similar to those of the elite athletes. She used her previous experiences as a *karateka*, researcher, and woman to inform her research-*doing* since the intersectional social issues faced by her and lived queer feelings motivated her research questions. She plunged into a process of self-reflection and counted on the guidance of the other authors to organise her learning in order to use it in her scholarship. That was, primarily, an experience of “practice” of subjectivity through examining others’ production of subjectivity, besides strengthening a positionality that lacked self-confidence. Thus, we explore issues around the researcher–practitioner theoretical–practical continuum of research-*doing*, presenting a journey that became empowering.

## 1. Introduction

Cartesian thinking perpetuates the mind–body dichotomy in several areas of society [1], expressed as binaries. When conducting research, we meet a binary as well, which disembodies the researcher as someone who is neutral, objective, and highly rational, while not letting feelings intrude [2]. We argue, though, that we are embodied beings, or *bodies of culture* as named by Resmaa Menaken, who are often not able to (and maybe should not) become detached from previous experiences and knowledge when conducting research. This can be a challenge, but we consider that a fluid non-binary positioning encompasses actions holistically and leads to tasks being performed on a continuum [3]. To do so, (self) reflexivity and critical thinking are required, which inform positionality, practice, and the review of theories and privileges [4].

We approach critical thinking and reflexivity following Freire’s [5] work, who advocated for the achievement of awareness of oppression in social matters in order for people to feel empowered and fight for freedom. He proposed that education would be the main tool in the achievement of (some) liberation, and this education plays a central role in empowering people to think freely. Therefore, people should become critical thinkers by reflecting on their own lived unjust experiences [6,7]. Such embodied critical reflexivity cannot be put aside but is embedded in people’s *doing* or practice, which, in turn, may ask for reviews in positionality [8]. We consider that positionality is not fixed, but also a continuum, requiring the acknowledgement of privileges to inform an honest standpoint [9]; yet, for the same purpose, theories may be revisited, and not taken as rigid norms, in order to provide a two-direction improvement for both theory and practice.

The purpose of this paper is to explore the reflexive process embedded in carrying out a PhD project committed to studying the production of the embodied subjectivities of a group of women high-level athletes in karate. Here, we focus on the experience of the researcher and first author of this paper who used excerpts of personal experience as autoethnographic material complementing the ethnographic contents from the high-level squad researched. This researcher was not an elite-level athlete; however, she was an experienced *karateka*, or karate practitioner, who has had several experiences competing at the amateur level in different countries, training in different *dojos*, or martial practice locations, and karate styles, and who faced experiences that were, to some extent, similar to those of the high-level athletes. She used her previous experiences as a *karateka*, researcher, and woman to inform her research-*doing* since the intersectional social issues faced by her and lived queer feelings [10], to some extent reported here, motivated her research questions.

Feelings of discomfort and inadequacy in environments served as a sort of compass for understanding other *karateka* women’s feelings. The researcher–*karateka* plunged into a process of self-reflection during the realisation of her PhD and counted on the guidance of the other authors in supporting her learning in order to use it in her scholarship. That was, primarily, an experience of “practice” of subjectivity through examining others’ production of subjectivity [11], besides strengthening a positionality that lacked self-confidence. Even though the PhD study followed a golden thread on gender and the conflicts faced mainly due to the binary order of gender, we emphasise here the binary (woman) scholar–practitioner, advocating for an embodied (holistic) conception (for our work focusing on the gender binary in karate, please see [12,13]). Thus, we explore issues around the researcher–practitioner theoretical–practical continuum of research-*doing*, presenting a journey that became empowering.

We start by briefly describing the methods we followed. In the next session, we present data in an integrative process of discussion and reflection, which is ongoing and (self) critically advancing. In this sense, we approach the first topic on *Critical reflexivity and continuum self-learning*; after that, we proceed to present *Queer feelings: uncomfortably estranging the comfortably familiar*, and we finalise with some concluding thoughts, though they are not fixed or straight, since the only permanent thing seems to be a changeful condition on *Repositioning: embodied subjectivity as permanent practitioner–researcher construction*.

## 2. Methods

We conducted a study in which the first author was undertaking her PhD degree. As people usually study what they have or what they lack, the PhD proposal basically departed from a personal need to verify if issues faced by the researcher–*karateka* were faced by other women *karateka* at other levels of the sport and in other countries considered to be more developed than that of the first author, who is from South America. Then, she took a journey seeking to discover, through an ethnography, how elite-level European women *karateka* preparing to take part in the first and unique, to date, Olympic Games that included karate, Tokyo 2020 (2021), dealt with challenges to build their embodied subjectivity. Since karate is among the sports (and yet as a martial art) that are understood to be masculine and of male preservation [14,15], women fighters usually need to battle in various and simultaneous fronts to reach space and respect, dismantling prejudice and discrimination.

The researcher–*karateka* moved to Spain to undertake her PhD and focused her study on the national Olympic squad, complementing the research yet in other two European countries, Scotland and Italy, and carrying out two foreign research internships. It was with the advent of COVID-19 that the implementation of autoethnographic pieces became a need, once the pandemic limited the initial plan for broad ethnography and forced adaptation (for more information, please see [16,17]). The autoethnographic data processing was conducted together with the ethnographic data provided by athletes through interviews, video analyses, and observations. The autoethnographic data from the first author was included and, at times, crossed with the elite athletes’ data in order to corroborate or better explain what was being said. The data set was organised into categories and shared with the other authors as well, who are experienced scholars and acted in a manner of triangulation of academics, to assure trustworthiness. However, in this paper, we are concentrating on the autoethnographic and critical reflexive experience of the first author; therefore, we are not bringing data from athletes and coaches, which can be found in our other publications, but unedited autoethnographic data. Considering this, it is relevant to mention a task and resource that helped to develop reflexivity, which was weekly blogs written by the first author to the third author.

In the internship in Scotland, the third author requested weekly writing in English for a free-themed blog of 200 words, expanded to 500 words due to the prolixity of the first author. Blogs were written for a number of pedagogical purposes, and not for research, and are a feature of the third author’s supervision of all of his doctoral students. They became, for the first author, a space for reflection on different themes, but especially related to the ethnographic experience, whether to reflect on lived experiences or on the theoretical framework. Many of the blogs helped the first author to come up with ideas that would be covered and expanded in the thesis. The weekly blogs exercise was continued beyond the completion of the PhD degree and during post-PhD as well. Writing blogs was a task that would initially take four months (the initially intended duration of the internship in Scotland) and ended up taking four years due to the first author’s choice and the agreement of the third author. The weekly blogs numbered 205 in total.

Next, we present a sub-session with theoretical information about autoethnographies in order to support our methodological path taken before addressing the central analysed part of our paper.

### Autoethnography

There are several studies using autoethnography as a source of reliable information for scientific research in social sciences. We may share some of them. Standal and Bratten [18] relied on autoethnography, with the second author researching her role as a teacher in a specific course that she was developing on physical education in Norway. As her proposal was articulated around embodied self-knowledge, she departed from knowing for herself how the movements were experienced and felt, thus knowing how they would arrive and be perceived by students. Standal and Bratten understand that the autoethnographic researcher “aims to elucidate cultural and social features of the context in which the self is embedded. As such, this research method is not a form of introspection or auto-biography, although both of these are a part of the auto-ethnographical method” (p. 3).

Landi [2], in turn, considers that the concept of objectivity, in scientific terms, is impossible to maintain, and argues that autoethnographies “challenge the objectivity of a silent researcher” (p. 6). He explains that this is not an exercise of egocentrism, but argues that the research process, as it goes through the researcher, is personal:

When we consider the ‘body-as-assemblage’ that is entangled with matter, meaning, and being, we recognise that all research is subjective and affective. (…) This autoethnography, a reflection of self-experience, is one way to disrupt the notion of ‘objectivity’, by using highly subjective narratives to offset the ostensibly objective nature of research.

Sparkes [19] writes about his task of performing evaluations of autoethnography papers. He clarifies by quoting Adams and Herrmann that autoethnography needs to comply with three dimensions, “‘auto’, ‘ethno’, and ‘graphy’. Thus, autoethnographic projects use selfhood, subjectivity, and personal experience (‘auto’) to describe, interpret, and represent (‘graphy’) beliefs, practices, and identities of a group or culture (‘ethno’)” (p. 290). He also addresses confusion often occurring between autoethnography and “confessional tales”, something that Delamont [20] also refers to, which we will return to later. He names different kinds of autoethnography, among which our research tends to transit between analytic and critical autoethnography. Taking inspiration from Anderson, Sparkes [19] explains that

the purpose of analytic autoethnography is not just about documenting personal experience, providing an insider’s perspective or evoking emotional resonance with the reader. Rather it is about using empirical data “to gain insight into some broader set of social phenomena than those provided by the data themselves”.

(p. 293)

By quoting Boylorn and Orbe, Reed-Danahay [21] describes critical autoethnography “as incorporating three aspects of critical theory: ‘to understand the lived experiences of real people in context, to examine social conditions and uncover oppressive power arrangements, and to fuse theory and action to challenge processes of domination’.” (pp. 144–145).

Finally, it is important to mention Loic Wacquant [22], who, within martial arts and combat sports, is considered a reference, developing his ethnography and autoethnography in Chicago boxing. Although martial arts ethnographies have older records, he inspired many studies that continued to find relationships between sport and Bourdieusian concepts. Sports ethnographies are rich in establishing several interdisciplinary relationships, often working with a broad spectrum to understand a specific topic in depth [23,24,25]. Having argued on the use of autoethnographic methodology, we proceed to present the main part of the article. We explain that to give the reflexive process the individual and personal character that it usually has, despite being built on the contribution of other authors and people, we will write in the first person singular from now on, returning to plural only in our concluding thoughts.

## 3. Critical Reflexivity and Self-Learning Continuum

A woman.
A *karateka*.
A scholar.
Yes. But…

When writing my PhD thesis [16], I reflected on my positionality and, at that point, I positioned myself in a sort of hierarchical order of different facets of myself. I stated the following:

I express myself primarily from my social place as a woman, that is, I do not see myself able to abandon my gendered subjectivity and generate and then read the data from a “neutral” point of view. After that, I consider my expression to be that of a researcher, finding contexts strange and with a strong interest in social problems. Finally, my expression as/of a *karateka* is presented, with some knowledge and mastery of the modality that allows me to capture internal elements of it.

(p. 64)

It is true that if a researcher is too identified with their topic, lacking the ability to distance [26] themselves from the lived experiences of a practitioner, their writing may be under the risk of becoming a propaganda of the topic more than critical scholarship. However, being too fragmented as in the quote is also not desirable, I would currently say. Such repositioning of myself may be justified due to the affect [27] of the very PhD research on myself. Undeniably, my subjectivity and lived experiences to date had influences on my reading of the data, and in a two-way street, the research itself exerted an influence on my being, affecting my way of reading the data and, maybe, even life, therefore in a mutual affectation process [28,29,30]. But this might depend on the topic of research. Gross [31], for example, in her experience as a scholar–practitioner, reports how she became a feminist before starting to publish. She also explores the idea of being an insider and outsider at the same time, “and therefore, in some senses, neither an outsider nor an insider” (p. 233), which adds to the continuum approach.

Thus, I would reformulate my own statement since I am in a permanent critical reflexive process, which has led me to envision things as not so separated, but on such a continuum that mixes several elements, although making things far more complex. Putting things in layers can be helpful for the explanation, but also carry the risk of Cartesian tendency being, then, a tramp. There is the risk of disembodiment, addressing things as “black or white”, as is usually said, without considering the umbrella of tones contained between these two colours. The very writing can lead us to see things as, to some extent, *broken*, in stages, following steps. Despite there being a process behind things, it is a continuum to be taken in its entirety. In this sense, art could be more effective in explaining what I intend in this example. Through a drawing, picture, painting, or sculpture, we can capture simultaneous symbols matching together, despite it having been built in a determined sequence. Therefore, my personal positionality remits to the ingrained idea of

A woman, scholar, and *karateka*, but…
Altogether.

I have embodied my research practice to an extent that I am always training and attentive to events, *strangely* discovering, and keeping an observer eye on every training session. If compartmentalising facets, as a researcher, I cannot be completely theoretical regarding my topic of study since I know the experience in and on my own flesh [32] in training sessions. My practice, viscerally lived, affects my standpoint as a researcher, and I do not intend to detach from that; on the contrary, I think that keeping it is positive once it attributes concreteness to analysed matters. It is about to “embody the science practice integration”, as in a scholar–practitioner experience reported by Holloway [33] (p. 6). For example, it gives me practical knowledge of what it means to be hit (punched or kicked), under the gender binary order, by a woman and by a man. Such a carnal experience makes me advocate for equity on several occasions to complement equality claims. Thus, there is, in place, the carnal sociology named by Wacquant, since what I experience through my body finds meaning when crossed with theories that explain feelings and situations of all orders lived on the mat.

This process in itself shapes the embodied subjectivity of the researcher–practitioner, considering that the practitioner self affects the researcher self and vice versa in a perpetual continuum. Personally, (lived issues in) my practice led me to carry out research. Notwithstanding, even as a child or teenage practitioner, I was always wondering about what I was being told by *senseis*, the graduate teachers or coaches. I believed them and embodied their teachings almost with faith, but I always reflected on such teachings and events, sometimes not exactly when they were happening, though. Was that a seed of a researcher? Possibly. In a similar manner is the work of Hynes [34], who delves into *Coghlan*’s experience as a scholar–practitioner taking *inquiry as a way of being* for performing action research. Nevertheless, I dare to say that reflexive critical thinking is what makes the affectation emerge [5]. Lived transformative experiences create knowledge according to the scholar–practitioner background of Navarro and Mistretta [35]. It is when we are reflexive that we make connections, that theory can explain practice, and practice can attest to theory. Maybe if we just stand for practice, there will be common sense in Gramscian terms; on the other hand, only theory could fall into idealism. Both of them together, more than intellectual or practical knowledge apart, would culminate in a sort of wisdom, as lived, applied, or tested knowledge. So, the *binomial* theory/practice becomes explained reality.

## 4. Queer Feelings: Uncomfortably Estranging the Comfortably Familiar

Some of my thoughts and feelings, embodied and *read* through my often-tense body, when disclosed here, lead to the challenge that showing vulnerability supposes. This is undoubtedly an uncomfortable task but also a humanising one, allowing people to know others to a point that brings them closer by discovering similar or shared issues, views, and perhaps experiences of “orphanhood” [36]; also in [10]. On this matter, justifying the experience of discomfort that may lead to action, I wrote the following in the thesis [16]:

Discomfort, or even the feeling of orphanhood, indicates the capacity for non-conforming or breaking the established, which makes it essential and almost appreciated. Appreciated when efforts turn to change, perhaps. Along these lines, all the suffering already experienced by so many women can assume a redemptive function, that is, it gains meaning, ceases to be in vain, and becomes justified. It is still suffering, I mean, obviously better if it could have been avoided. But the cause of acting to avoid possible future suffering, known in one’s own skin, is, precisely because of this empathic knowledge of how much it hurts, a powerful driving force for change.

(p. 306)

There is perhaps a way of finding relief from the need to face adversity. It does not mean that I would advocate for toughness in life in a stoic mode, claiming that only hard work can be dignifying and that traditional old-fashioned approaches would be the best. This is not the path I take [16]. However, when, for different reasons, you cannot control life events, adopting a sort of open serendipitous perspective [37] can be helpful to overcome challenges and extract strength from difficulty. It may sound too idealistic when we are immersed in life’s trials, but the capacity of being resilient and convert suffering from adversity into an engine for transformative political action [31,36,38] can, in itself, become a source of ethical care [39] for oneself and others, ending up in a (started, at least) healing process. It does not imply, at all, that social justice will no longer be pursued.

Another point to mention on self-exposure relates to the critique made by Delamont [20] on autoethnographies when “It is about me and my introspective emotions and my personal life” (p. 57). For her, it is necessary to differentiate “productive and unproductive uses of reflection versus autoethnography” (p. 57). Her work led me to reflect on the reliability of thoughts and feelings brought to the autoethnographic setting of my research. Can *this* be science and relevant work? I wondered. After arguing with myself, I would say that using critically reflected experiences that expose vulnerability in an uncomfortable way in order to advocate for the change in unfair situations, and not to promote oneself, is committed science and relevant work, as has been shown by other scholar–practitioner researchers [31,40] or autoethnographers [41].

Notwithstanding, achieving the strength and confidence to make this previous statement required a path. Reflecting on such a path, I would say that the first step is, perhaps, the work on building confidence, which, unfortunately, is not completely reached in my case. I believe I share an embodied lack of confidence with several other women, scholars, and *karateka*, who unfortunately often need to deal with imposter syndrome [42]; on lack of confidence, see [12,43]. Some examples of thoughts of not fitting in and inadequacy, and at the same time, inadequate thoughts on the good and health of oneself, could be the following:

This is not my world.
What am I doing here?
I will never find my place.
Who do you think you are, Fabiana?
There is no solution for me.
Why do I feel so dislocated?
I’m so stupid.

Yet, in agreement with the work of Young [43], the social construction of girls and women imbues us with a sort of inferiority feeling, so we tend to be adaptive to the male gaze [44], often feeling insecure and seeking external approval. This relates to the patriarchy, but also to other intersectional discriminations. Issues faced by me on ethnicity and social class, for example, play an important role in my lack of confidence. Even though one can decide to extract the best of bad situations, e.g., making good use of queer feelings that teach us how to be empathetic, it is still about pain. And if it transcends the border of discomfort to discrimination, it then turns into a matter of social justice, requiring action and crossing boundaries [31]. The first way to deal with a lack of confidence, therefore, would be, through reflexivity and critical thinking, to become aware of social injustices and manipulations carried out in the name of power and domination [5].

Remaining in discomfort is not a sustainable pursuit. So, after becoming aware of problems, in a salutogenic approach [45], there is a need for action. There are different levels of action depending on the issue. An initial and highly important issue relates to finding support. In the specific situation I have been exposing here, I was lucky enough to meet supportive supervisors. I acknowledge a third supervisor of my PhD, who does not author this paper with us but remains admired and respected by me. The second and third authors of this paper, supervisors of my PhD degree as well, have continuously been supportive of my work and of me in an embodied meaning. This helped to make the journey truly empowering. I share an excerpt of a personal blog that reported this process:

I have read lots of texts. (…) They enable knowledge, eliminate superficial beliefs and *produce power*. Knowledge is, in fact, liberating. I am immersed in several theories with the writing of the thesis and the fact is that I like it very much. Somehow this, the theories themselves, the feeling of approaching some knowledge that I have always feared —I had never read Butler, for example, out of fear; I was always afraid and found it complex—, it contributes to a process of empowerment. Many theories are, in fact, quite complex, but I think now that I have capacity to understand them. I hope I am not surprised otherwise in defending the thesis! So, what I want to express is that I feel a little more confident than I used to be. (…) I was thinking and even idealizing that at some point I can generate academic contributions that may also empower other women.

[46]

I am conscious of the critique I am open to once I am reporting support received from an entirely men supervisor team. Despite the privileges they will always hold in patriarchal societies, undeniably, there are respectful and aware men working with and in favour of women. Keeping in mind the umbrella of genders between the binary women–men, women need to work in partnership with other genders in order to reach better conditions of living and social justice. I make a quick point here to be coherent with the critique I am posing of binarism. I acknowledge the umbrella of genders we have and advocate for the celebration of difference. Nevertheless, I am referring to “men” and “women” in a generalisation [47] that follows my PhD research [16] carried out in the *karateka* environment, which is a strong gender binary environment. Also, the specific situation involving my supervisors and I respond to the given set of self-identified people as a cis-gender woman PhD student (altogether) and cis-gender men supervisors. The three of us recognise the privilege we dispose of in positioning as cis-gender people.

Regarding empowerment, it can take place both individually and collectively, making people more independent to act in the world to the point of emancipation [48]. Empowerment is undoubtedly related to power and the acquisition of it in its positive meaning, e.g., people who did not have access to forms of power and then gained power; resources, be it material or non-material, such as knowledge; and collaboration, like support, collaborative, and voluntary engagement [48]. Other features of empowerment might be a greater awareness of freedom, a challenge of gender stereotypes, self-esteem, self-determination, the acquisition and mastery of skills, and social benefits [49]. When people feel empowered in an emancipatory sense [5], they develop a critical understanding of themselves and the environments they attend to, which can be converted to even greater empowerment. People feel in possession of extra strength that drives them to fight for what they want, believe, and deserve, and they also have a greater capacity to maintain their achievements [48].

Bringing all of this to the context of a woman researcher and *karateka*, I think that the lack of fairness in society can make experiences uncomfortable everywhere, even when there is domain of a practice. Thus, the supposedly comfortable known place, with which one is familiar, may be converted into something strange, uncomfortable, or weird. Likewise, experiences of marginalisation, and of being left out, contribute to making queer feelings known [10,50]. Then, even when there is some mastery in place, for example, the embodiment of and compliance with criteria to be a member, a black belt, an athlete holding results, and so on, there is still unfamiliarity within the known. This is again about the fluidity between the insider–outsider positions [31], or an *outsider within* [51]. There is a set of complex contradictions, which are unable to deny trustworthiness, though they are made of continuous strangeness of *belonging* to environments that may not feel properly secure. On the other hand, sometimes there is also the contradiction of a kind of familiarisation with the discomfort, or some adaptation to unpleasant contexts to which one should complain about and rebel against.

Usually, people who are feeling uncomfortable leave environments where they do not feel as welcomed [52]. Notwithstanding, this could be a matter of empowering people and challenging environments, taking into account that empowered people may change environments. Such a change can be obtained through queering attitudes and moments [6], with people queering their own embodiment in different but mixed-up facets of life, like in the case of a *karateka*, woman, and scholar. According to Phipps [53], the queer theory developed out of the gay liberation movement that followed the 1980 AIDS crisis, as many clashes were raised against gay people. lisahunter [54] (p. 2), though, considers that

Queer theory emerged from a foundation of several origins and influences including, but not limited to, activist and academic iterations of feminism, lesbian and gay movements promoting political transformation of recognition and rights for diverse sexualities, poststructural and postmodern theory, critical theory, radical race theory, postcolonial theory, disability and transgender studies. Normative notions of sex, gender and sexuality along with assumed relationships between the three, and a critique of identity categories and their markers resulting in social difference were all targets of queer studies and queer theory.

Queer etymologically means “twisted or crossed” [55] (p. 129). lisahunter [54] explains that “queer theory seeks to dismantle categorical notions, challenge the heteronormative perspective, and move beyond sex, gender and sexuality categories” (p. 1). This conception is relevant because it expands some boundaries and does not place theory as being under the possession of just a few people [56]. Perhaps queerness can be lived in the experience of inadequacy, discomfort, and maladjustment to different situations [10]. Feeling strange, weird, oblique, and out of place can be configured as the necessary baggage to understand what is queer [2]. Mock [56] (p. 20) says that “‘Doing’ queer means possessing the agency to defy and destabilize gendered behaviours, sexes, and sexualities through continuing and conscious decisions.” lisahunter [44] (pp. 2–3) also contributes by exposing that

Unsettling assumptions, challenging the work and outcomes of normativity, revealing oppressions associated with categories, exposing essentializing identities and subject positions, creating heterogeneous and fluid identities, stripping categories of their naturalness, decoupling sex/gender/sexuality, stimulating relationships beyond androcentric notions of able bodies, etc. is all part of doing queer work, or queering.

## 5. Concluding Thoughts

### Repositioning: Embodied Subjectivity as Practitioner–Researcher Permanent Construction

The purpose of this paper was to explore the reflexive process of the primary researcher of this paper when carrying out her PhD project. While she studied the production of the embodied subjectivities of a group of high-level women athletes in karate, she was also learning [33] and repositioning herself regarding her own embodied subjectivity in a mutual affectation process [34]. The *karateka*–researcher took an investigative path motivated by her lived experiences, which kept her journey as viscerally embodied and favourably counting on her advocacy for a continuum instead of a binary in the binomial scholar–practitioner. Her lived queer feelings, obliquely felt and deeply reflected, worked as a compass in the empathetic exercise of understanding other *karateka* women’s feelings. Then, through empowering discoveries made by delving into the literature, especially feminist literature, uncomfortable feelings were converted into a potential path for change. Queering environments, positions, attitudes, or moments represent, then, a disruptive way of exerting agency over consolidated structures. This, in turn, provides evidence for us to argue in favour of another continuum, that of theory⇔practice, and the possibility of crossing boundaries in these terrains [31].

Considering this, we would like to comment on a transversal finding of the PhD in the conducting of interviews related and relevant to what was commented here. We conducted two interviews with each of the 18 participants and perceived how the athletes started realising issues in karate while they were reflecting to answer the questions. Maybe they were aware of some problems and shared them when they felt like they were in a safe space. But we also found moments in which they were repositioning themselves on matters that seemed just *to be like that*, meaning things that are assumed to be the way they are since they have become accepted simply as problematic and are not thought of as being capable of change. Such things, thus, are not even considered to be modified. So, there is a disguised form of domination in place to an extent that discourses and prejudices, which are ultimately harmful to people, end up embodied by the very people who should fight it. Even though such embodiment can, at times, be in a manner of joining the strong side, e.g., through the performance of hegemonic masculinities carried out by women, this is usually carried out in order to achieve belonging. However, independent of the reason, it seems to respond to disguised ways that power dynamics work and lead to the maintenance of convenient established structures.

Following this line, it was insightful to verify that women *karateka* in the study at times considered that they did not suffer discrimination in their journeys. Notwithstanding, this discrimination was re-elaborated in the course of the interviews. Sometimes they seemed to want to prove their worth by trying to equal men, and at other times, they just repositioned themselves when they became aware of problematic situations that they assumed to be normal before; they honestly repositioned themselves when they found a sort of welcoming space that was supportive and empathetic to their trials, allowing them to feel safe. Therefore, what we intend here is to advocate for the courage of repositioning, which emerges when reflexivity and critical thinking are stimulated and lead to awareness. There is no problem, on the contrary, in revising previous standpoints and redirecting positioning, which is in constant formation. This is because there are times when we may be somewhat naïve victims of contemporary issues that we face but take them as *normal* or simply *the way things are*. And this justifies our exercise of sharing vulnerability as a practice of empathy that can hopefully build bridges. This is not just about complaining, but about becoming strong together, caring about each other and others’ struggles, finding healing in becoming empowered, and, in doing so, changing unjust situations.

Finally, we would like to highlight the contributions we envision that our work may represent to the area of scholars–practitioners, especially from the point of view of a continuum, as we have been arguing. The binomial scholar–practitioner is not widely understood, and even less, taken as fluid or a continuum. We know that it remains as a binary instead, as much as others we brought here, such as gender order or theory–practice. The in dash (–) should fill the gap between the words, putting them together almost as one. But it does not, showing that the Cartesian model still prevails, as much as the resistance of some readers. With this work, though, we are proposing the crossing of some boundaries, maybe expanding traditional “boxes” in which people should fit to allow them to be served by systems. It is not about denying rigour to things that are done but being less oppressive and allowing for empowerment and beneficial fluidity in both scholarship and praxis.

## Data Availability

Due to privacy protection, the data of the informants of the PhD are not provided.
